# Integrated Proteogenomic Approach for Discovering Potential Biomarkers in Urothelial Carcinoma of the Bladder

**DOI:** 10.3390/biomedicines13123020

**Published:** 2025-12-10

**Authors:** Pongsakorn Choochuen, Surasak Sangkhathat, Wararat Chiangjong, Worapat Attawettayanon, Kittinun Leetanaporn, Komwit Surachat, Panupong Sukpan, Wararak Kaewrattana, Ornsinee Senkhum, Natthapon Khongcharoen, Natakorn Nokchan, Nifahmee Hayiniloh, Dussadee Nuktong, Pasu Tansakul, Kant Buaban, Anas Binkasem, Virote Chalieopanyarwong

**Affiliations:** 1 Department of Biomedical Sciences and Biomedical Engineering, Faculty of Medicine, Prince of Songkla University, Songkhla 90110, Thailand; pongsakorn.mwit20@gmail.com (P.C.);; 2 Division of Surgery, Faculty of Medicine Siriraj Hospital, Mahidol University, Bangkok 10700, Thailand; 3 Department of Pediatrics, Faculty of Medicine Ramathibodi Hospital, Mahidol University, Bangkok 10400, Thailand; 4 Division of Surgery, Faculty of Medicine, Prince of Songkla University, Songkhla 90110, Thailand; 5 Translational Medicine Research Center, Faculty of Medicine, Prince of Songkla University, Songkhla 90110, Thailand; 6 Medical Education Center, Naradhiwas Rajanagarindra Hospital, Narathiwat 96000, Thailand; 7 Department of Urology Surgery, Songkhla Hospital, Songkhla 90110, Thailand

**Keywords:** urothelial carcinoma, proteomics, transcriptomics, biomarker discovery

## Abstract

**Background/Objectives**: Urothelial carcinoma of the bladder (UCC) is a leading cause of cancer-related death globally. Given that urine is in direct contact with the tumor, it represents a highly valuable source for non-invasive molecular analysis. **Methods**: This study utilized liquid biopsies from 41 UCC patients and 27 non-cancerous hematuria controls to identify novel diagnostic and prognostic biomarkers via proteomic and transcriptomic analysis. **Results**: Urine proved to be a reliable source, yielding a mean tumor cell fraction of 0.605 (95% CI: 0.505–0.705). We identified 11 genes with concurrent alteration at both the urinary protein and mRNA levels. Notably, four upregulated markers, *CYTB*, *C1QC*, *SBP1*, and *ANXA4*, demonstrated strong diagnostic potential, with AUC values greater than 0.70. *CYTB* and *ANXA4* were detectable even in early-stage UCC (stages Cis, I, and II). Furthermore, we identified two proteins, CATC and SPB10, that were markedly upregulated in recurrent UCC and correlated with poor overall survival, positioning them as potential prognostic markers for recurrence risk. **Conclusions**: This study confirms the utility of urine as a reliable medium for detecting UCC tumor cells, offering promising markers for both early-stage diagnosis and predicting NMIBC recurrence.

## 1. Introduction

Bladder cancer (BC) is a substantial global health concern, ranking as the ninth leading cause of cancer-related death worldwide. Incidence and mortality rates among men stand at 9.5 and 3.3 per 100,000, respectively [1]. Over 90% of BC cases are urothelial carcinomas (UCC), while only 5% are squamous cell carcinomas [2]. Approximately 70–80% of BC cases are diagnosed as non-muscle invasive BC (NMIBC), with the remaining 20–30% classified as muscle invasive BC (MIBC) [2]. Although most patients are initially diagnosed with NMIBC, the recurrence rate for NMIBC is relatively high, ranging from 50% to 70% [3,4]. Additionally, 10–20% of NMIBC patients experience disease progression to MIBC, which reduces the 5-year overall survival rate to about 50%, even with intensive treatment [5]. Cystoscopy remains the standard method for diagnosing UCC and conducting long-term follow-up. However, its utility is constrained by its invasive characteristics and economic burden. Moreover, the procedure carries potential risks for the patient, including the induction of infection, pain, and iatrogenic hematuria [6]. Consequently, many recent studies have focused on using liquid biopsy as a new diagnostic approach to lower the risk of post-procedural complications [7].

In recent years, comprehensive omics studies have improved our understanding of the underlying molecular mechanisms and identified several biomarkers associated with UCC pathogenesis. Urine, which directly contacts the UCC tumor, is regarded as a promising source for detecting tumor cells [8]. Some of these markers have received approval from the FDA or European Conformity (CE) for clinical use. However, their usefulness is still limited due to their relatively low sensitivity or specificity compared to cystoscopy [9,10]. As a result, using urine markers alone is currently not recommended for bladder cancer screening and diagnosis [11,12].

In this study, we conducted proteomic and transcriptomic analyses of urine samples from patients with UCC and compared them with samples from patients with non-cancerous hematuria. Our goal was to uncover the underlying molecular mechanisms and identify potential UCC markers. We hypothesized that dysregulated proteins, resulting from the expression of their corresponding mRNAs produced by tumor cells, are secreted into the urine. These proteins could serve as indicators of UCC presence and might be relevant to patient outcomes. Additionally, we evaluated the diagnostic performance of these markers to assess their potential for clinical use.

## 2. Materials and Methods

### 2.1. Patient Recruitment and Data Collection

The tumors and urine samples used in this study were obtained from a group of 41 patients diagnosed with urothelial carcinoma. These patients underwent either transurethral resection or radical cystectomy at Songklanagarind Hospital, Prince of Songkla University. For the control group, normal urine samples were collected from 27 unmatched patients who presented with hematuria due to noncancerous causes. First-morning urine samples from both groups were collected on the day of the procedure between 2020 and 2023 and preserved with a Urine Preservative Single Dose (Norgen Biotek, Thunder Bay, ON, Canada). Primary tumor tissues were surgically removed and snap-frozen in liquid nitrogen for preservation. None of the patients in the cohort had previously undergone chemotherapy or radiotherapy for their respective diseases.

Clinical and demographic data for all patients were retrieved from the hospital’s electronic medical records (EMR). This information included demographics, histopathological details, treatment methods, TNM staging, and patient outcomes. The workflow of this study is shown in Supplementary Figure S1.

### 2.2. Urine Processing

The first-morning urine was processed using an in-house differential centrifugation method modified from Livshits et al. [13] to obtain cell-free urine and urinary cell pellets. Specifically, 50 mL of urine from each sample in both the UCC and control groups was centrifuged at 500× *g* for 10 min at 4 °C to separate the suspended cells. The resulting cell pellet was collected, and the supernatant was subjected to secondary centrifugation at 20,000× *g* for 30 min at 4 °C to remove debris and large vesicles. Pellets from both centrifugation steps were combined and used for total RNA extraction using the RNeasy^®^ Mini kit (QIAGEN, Hilden, Germany). RNA quantity was assessed using a NanoDrop 2000 spectrophotometer (Thermo Fisher Scientific, Waltham, MA, USA), and quality and integrity were determined with an Agilent 2100 BioAnalyzer (Agilent Technologies, Santa Clara, CA, USA). Cell-free urine obtained after secondary centrifugation was used for total protein extraction following an in-house protein precipitation protocol adapted from Afkarian et al. [14]. Specifically, 10 mL of urine was mixed with 30 mL of cold 100% ethanol to achieve a final concentration of 75% (*v*/*v*). The mixture was incubated at 4 °C for 15 min to precipitate proteins, followed by centrifugation at 14,000× *g* for 30 min at 4 °C. The resulting protein pellet was washed twice with 5 mL of 75% ethanol, air-dried, and subsequently dissolved in 100 µL of 1× Laemmli’s buffer (Bio-Rad, Hercules, CA, USA). Protein concentration was quantified using the Bradford assay (Bio-Rad, Hercules, CA, USA).

### 2.3. Tissue Processing

10–20 mg of tissue was dissected from fresh frozen tumors and used for total RNA extraction with a RNeasy^®^ Mini kit. The quality and concentration of the extracted RNA were subsequently assessed using an Agilent 2100 BioAnalyzer and a NanoDrop 2000 spectrophotometer, respectively.

### 2.4. RNA-Sequencing and Data Processing

RNA-seq libraries were prepared using total RNA extracted from both tissue and urine sediment. TruSeq Stranded Total RNA with the Ribo-Zero H/M/R_Gold kit (Illumina, San Diego, CA, USA) was used for library creation. Then, paired-end 150 bp sequencing was carried out on an Illumina NovaSeq 6000 platform (Illumina, San Diego, CA, USA). Library preparation and RNA sequencing were outsourced to an external service provider (Macrogen, Inc., Seoul, Republic of Korea). The RNA-seq analysis workflow was based on transcript-level expression analysis [15]. First, the quality of the raw RNA-seq data was checked with FastQC (v0.12.1) [16]. Trimmomatic (v0.39) [17] was used to remove adapters and low-quality sequences. The reads were aligned to the human reference genome (version GRCh38.13) with HISAT2 (v2.2.1) [18]. The resulting Sequence Alignment Map (SAM) files were converted into Binary Alignment Map (BAM) format, sorted, and indexed using SAMtools (v1.17) [19]. Samples with an overall alignment rate above 75% were included in further analysis. Transcript assembly was performed using StringTie (v2.2.1) [20] to reconstruct transcripts from the aligned RNA-seq reads. The assembled transcripts from each sample were then merged using StringTie to create comprehensive transcript annotations. These merged transcripts were used as reference annotations to re-estimate gene expression in each dataset sample.

### 2.5. Inference of Tumor Purity and Microenvironment

We estimated the fraction of tumor cells in the cancer samples using ESTIMATE [21]. Additionally, we employed xCell [22] to determine the proportions of stromal, immune, and microenvironment cells in both cancer and control samples. The fraction of each cell type was measured using xCell scores, which included ImmuneScore, StromaScore, and MicroenvironmentScore. Differences in these xCell scores between the sample groups were statistically compared using the Wilcoxon rank-sum test, with significance set at *p* ≤ 0.05.

### 2.6. Proteomic Sample Preparation and Proteomic Data Acquisition

A 50 µg aliquot of protein from each sample was subjected to an on-column trypsin digestion method. Proteins were initially denatured using 8 M urea in 50 mM Tris-HCl (pH 8). Reduction was carried out with 5 mM dithiothreitol (DTT; Sigma, Burlington, MA, USA) for 1 h at 37 °C, followed by alkylation with 15 mM iodoacetamide (IAA; Sigma, Burlington, MA, USA) for 1 h at room temperature in the dark, under agitation. Proteolysis was performed using trypsin and LysC (Promega, Madison, WI, USA) at a 1:50 enzyme-to-substrate ratio for 20 h at 37 °C with gentle mixing. The reaction was quenched by acidifying the solution to pH < 3 with 5% formic acid (FA; Merck, Kenilworth, NJ, USA). Peptides were subsequently desalted and purified using C18 solid-phase extraction (SPE) disks (EMPORE™-3M, Cottage Grove, MN, USA) and C18 beads, following a protocol adapted from Rappsilber et al. [23]. The resulting dried peptides were resuspended in 0.1% formic acid to a final volume of 50 µL, yielding a concentration of 1 mg/mL.

Urine peptides were analyzed individually in a single batch using the Sequential Window Acquisition of All Theoretical Mass Spectra (SWATH) method, which integrates data-independent acquisition (DIA) with spectral library matching [24]. All urine protein samples were measured individually within the same batch and run on the same capillary column. Initially, 2 μg aliquot of peptides was injected into an ultra-high-performance nanoflow LC system (Eksigent, Dublin, CA, USA) equipped with a C18 trap and analytical column (Nano Trap TP-1, 3 µm, 120 Å, 10 mm × 0.075 mm; bioZen Peptide Polar C18 nanocolumn, 75 µm × 15 cm, 3 µm particle size, 120 Å; Phenomenex, Torrance, CA, USA). Elution utilized a 105 min gradient, with Solvent A being 0.1% formic acid in water and Solvent B being 0.1% formic acid in acetonitrile (ACN). Mass-to-charge (*m*/*z*) data were acquired using a TripleTOF 6600+ instrument (ABSciex, Toronto, ON, Canada) in positive ion mode SWATH-MS/MS. The eluate was ionized via an OptiFlow Turbo V Source (ABSciex). SWATH scans utilized a precursor mass tolerance of 100 ppm and a fragment mass tolerance of 0.2 Da within a detection range of 300–1800 Da. The DIA mode employed a 7 *m*/*z* window with a 1 *m*/*z* overlap. All raw data (Wiff file) were processed for protein identification and quantification using Protein Pilot v.5.0.2.0 (ABSciex), referencing the Swiss-Prot database (UniProtKB 2022_01) for *Homo sapiens* (20,385 proteins). Search parameters included IDA Carbaminomethyl (C) as a fixed modification, trypsin-lysC digestion, allowance for one missed cleavage, mono-isotopic mass, and a false discovery rate (FDR) threshold of less than 1%. For DIA analysis, 10 min extraction windows, 25 peptides per protein, and six transitions per peptide were used; shared peptides were excluded, with a 20 ppm XIC width and an FDR cutoff of less than 1%.

### 2.7. Transcriptome and Proteome Data Processing

All data processing steps were performed using the statistical language R (v4.3.2). The gene expression matrix was generated with the ‘IsoformSwitchAnalyzeR’ package (v2.0.1) [25], while protein expression data was obtained from Protein Pilot software (relative protein abundance available in the Supplementary Material). Gene counts were normalized using the fragments per kilobase of transcript per million mapped reads (FPKM) method. The protein expression levels were normalized with a log2 transformation followed by median subtraction. Principal component analysis (PCA) of the transcriptome and proteome data was carried out using the PCAtools package (v2.16.0). The normalized gene and protein expression data served as input. Correlation analysis between urine and tissue mRNA expression was conducted using Spearman’s correlation test. *p*-values and correlation coefficients were calculated, and the results were visualized with a scatter plot.

Subsequently, differential gene expression (DGE) analysis was performed by comparing the UCC and control groups using the edgeR package (v3.32.0) [26]. For the proteome data, differential protein expression (DPE) analysis was conducted using the limma package (v3.56.2) [27] to examine the log2-fold changes in protein levels between urine samples from the UCC and control groups. To screen for potential markers to be included in further validation, a significance threshold of *p* ≤ 0.1 was applied.

### 2.8. Network and Pathway Analysis

Pathway and process enrichment analyses were performed using Metascape [28]. Terms were selected based on strict criteria: a *p*-value ≤ 0.01, a minimum gene count of 3, and an enrichment factor greater than 1.5. Selected terms were grouped into clusters based on their membership similarities. Specifically, *p*-values were computed using the cumulative hypergeometric distribution, and q-values were calculated to correct for multiple hypothesis testing. The kappa score was used as the similarity metric for hierarchical clustering of the enriched terms. Sub-trees exhibiting a similarity score exceeding 0.3 were defined as clusters, and the most statistically significant term in each cluster was chosen as the representative term.

A network plot was constructed to visualize the relationships among the enriched terms. In this network, edges connected terms with a similarity score above 0.3. For visualization, a subset of terms was chosen: the top terms with the lowest *p*-value from each of the top 20 clusters, ensuring a limit of no more than 15 terms per cluster and an overall maximum of 250 terms. The final network was rendered using Cytoscape 5 (v3.10.2) [29].

### 2.9. Identification of Biomarkers

Pathway urine proteins that showed significant dysregulation at both mRNA and protein levels were prioritized as candidate markers. Afterwards, we assessed the expression of these selected candidates in early-stage UCC by conducting protein differential expression analysis and comparing urine proteomic profiles between patients with early-stage UCC and a control group.

To identify prognostic markers, we examined the association between protein expression and recurrence in patients with NMIBC. We prospectively monitored recurrence events in our cohort over one year following surgery, dividing patients into recurrent and non-recurrent groups based on these events. Recurrent events in our cohort tended to occur around three months post-surgery, indicating that the risk of recurrence due to incomplete resection is relatively low [30]. Markers with a *p*-value ≤ 0.1 were preemptively considered differentially expressed. Additionally, we conducted further analyses on these candidate markers to assess their diagnostic potential using publicly available proteomic profiles as validation datasets.

### 2.10. Evaluation of Protein Marker Potency with Public Databases

In our study, we obtained the validation dataset from publicly available proteomic data through a systematic search. Specifically, we explored the ProteomeXchange Consortium [31], which encompasses six major global proteomic repositories: PRIDE, PeptideAtlas, MassIVE, jPOST, iProx, and Panorama Public. Our search aimed to identify relevant proteomic datasets published from 2015 to 2024. The keywords used were “bladder cancer” and “proteomic.” Through this approach, we identified a total of 14 relevant datasets. We screened these datasets based on inclusion and exclusion criteria. Of the initial 14, we excluded 11 datasets for various reasons: (1) Lack of Global Proteomic Profile: Five datasets lacked comprehensive proteomic profiles of normal bladder tissue or urine samples. (2) Post-Chemotherapy Proteomic Profiles: Three datasets contained only proteomic profiles specific to post-chemotherapy bladder cancer. (3) Extracellular Vesicle-Derived Profiles: Two datasets were derived solely from extracellular vesicles, which did not align with our study objectives. (4) Glycoproteomic Profile Only: One dataset provided only glycoproteomic profiles, limiting its utility for our validation analysis. Following this rigorous screening, we narrowed down our selection to three datasets. However, upon closer examination, two of these three datasets lacked sample datasheets or data labels. Consequently, we chose the remaining dataset with the project ID PXD010260 as our validation dataset for further investigation.

Public proteomic data from project ID ‘PXD010260’ was obtained from the ProteomeXchange Consortium database [32]. Proteomic data were derived from eight NMIBC tissues and eight matched normal bladder tissues. Among the eight patients, three experienced recurrent disease prospectively. Before data analysis, protein expression data were log2-transformed and used as a validation dataset. To assess the diagnostic potential of the candidate protein markers, we used logistic regression to build a prediction model for each marker. The protein expression levels from our cohort served as the training data, while the sex and age of the patients were included as independent variables. The resulting model was then applied to predict the probability of disease occurrence in the validation dataset. The diagnostic ability of the urine biomarkers, reflected in the probability of disease occurrence, was evaluated using a receiver operating characteristic (ROC) curve and its area under the curve (AUC). The optimal cut-off value, calculated from the maximum Youden’s index during ROC analysis, was used to determine the sensitivity and specificity of each marker.

To verify the potential of the protein marker linked with disease recurrence, eight patients diagnosed with NMIBC from the same project were divided into two groups: recurrent and non-recurrent. Differential expression analysis of proteomic data from these groups was conducted to identify proteins associated with recurrence. The results were compared with findings from our initial cohort. To assess the relationship between the identified recurrent marker and clinical outcome, a survival analysis was performed using the Kaplan–Meier method to evaluate survival probability, and the log-rank test was used to compare survival rates. The software tool used for this analysis was the Kaplan–Meier Plotter (http://www.kmplot.com; accessed on 25 May 2024) [33].

### 2.11. Statistical Analysis

Statistical analyses of clinicopathological features were conducted. Ordinal variables were compared using the Wilcoxon rank-sum test, while categorical variables were compared with Fisher’s exact test. All statistical tests were two-sided, and a *p*-value ≤ 0.05 was considered statistically significant.

## 3. Results

### 3.1. Clinical Characteristics of the Patients

**Figure 1:**
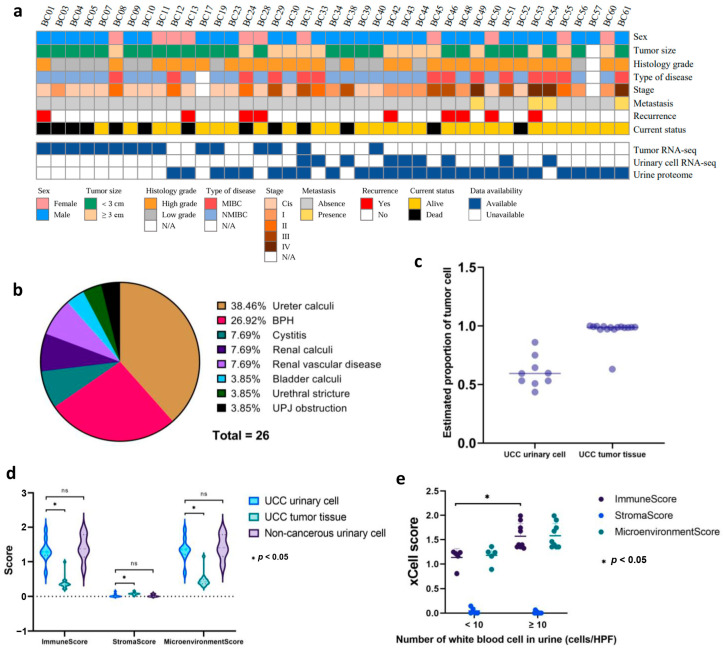
Clinicopathological features and urine cellularity of patients (**a**) Heatmap representing the clinicopathological attributes of 41 patients with UCC. Various clinicopathological traits are denoted by distinct color codes. (**b**) The pie chart illustrates the proportion of the control group based on the etiology of non-malignant hematuria. (**c**) Estimated proportion of tumor cells in both urinary cells and tumor tissue using ESTIMATE. Each dot represents the proportion of tumor cells in each sample. (**d**) Estimated proportion of immune and stromal cells in UCC, control urinary cell, and UCC tissue as indicated by xCell score. ns means non-significant. (**e**) Association between hematuria severity grades in the control group and their corresponding proportions of immune and stromal cells.

We initially recruited 41 UCC patients and 27 patients with non-cancerous hematuria as the control group. We collected first-morning urine samples from patients in both groups. Additionally, we obtained cancer tissue samples from 15 UCC patients. Figure 1a and Supplementary Table S1 display the clinical characteristics of all patients. In the UCC group, 73.2% were male, and 26.8% were female, consistent with the global sex-related incidence of UCC [4]. The median age at diagnosis was 74 years (range 42–94) for the cancer group and 64 years (range 50–89) for the control group. Among the 41 UCC cases, 61.0% were classified as NMIBC, and 34.1% as MIBC. Regarding histological grading, 68.3% had high-grade histology, while 29.3% had low-grade histology. In terms of COG stage, 39.0%, 19.5%, 12.2%, 14.6%, and 9.8% were classified as stages I, II, III, and IV, respectively. The proportions of patients based on histological grading, disease type, and stage aligned with previous reports [34]. Additionally, nine patients (22.0%) in the UCC group experienced recurrent disease following primary surgery. In the control group, ureter stones were the most common cause of hematuria, accounting for 38.46% of cases, followed by benign prostatic hyperplasia (BPH, 26.92%). The remaining 34.62% of hematuria cases were caused by cystitis, kidney stones, renal vascular disease, bladder stones, urethral strictures, and ureteropelvic junction (UPJ) obstruction (Figure 1b).

Out of the total 68 cases from both groups, we performed urinary RNA sequencing on 9 UCC cases and tissue RNA sequencing on 15 UCC tumors. To serve as controls in our transcriptomic analysis, we also conducted urinary RNA sequencing of 7 patients with non-cancerous hematuria. The median RIN scores for UCC urinary cells, control urinary cells, and cancer tissue were 4.4 (interquartile range (IQR), 1.80–5.05), 2.4 (IQR, 1.70–4.50), and 7.2 (IQR, 6.0–7.8), respectively. In a subset of 53 cases, including 27 UCC cases and 26 controls, we performed comprehensive global proteome profiling (Figure 1a). Statistical comparison showed no significant difference in clinicopathological characteristics between the cancer and control samples in each transcriptomic and proteomic analysis (Table 1).

### 3.2. Urinary Cellularity and Purity

The proportion of tumor cells in each sample was estimated using the ESTIMATE tool. The results showed that all urinary cell samples in our cohort had sufficient neoplastic purity, with an average tumor cell proportion of 0.605 (95% CI: 0.505–0.705) compared to tumor tissue, which had a very high average tumor cell proportion of 0.963 (95% CI: 0.913–1.000) (Figure 1c, Supplementary Table S2). Furthermore, we used xCell to analyze the cellular components within the tumor tissue and urine samples from both the UCC and control groups. The findings indicated that the tumor tissue had a higher median proportion of stromal cells (0.076, 95% CI: 0.055–0.097) than urinary cells from the UCC group (0.000, 95% CI: 0.000–0.039) and the non-cancerous group (0.000, 95% CI: 0.000–0.029). In contrast, urinary cells from both groups showed a higher proportion of immune cells, as reflected by the elevated median ImmuneScores of 1.285 (95% CI: 1.020–1.550) for UCC urinary cells and 1.372 (95% CI: 1.024–1.721) for non-cancerous urinary cells, compared to UCC tissue at 0.346 (95% CI: 0.246–0.447) (Figure 1d). It is worth noting that urine samples from both groups were collected from patients with hematuria. The comparison demonstrated that patients with higher hematuria grades (WBC ≥ 10 cells/HPF) had significantly higher median ImmuneScores of 1.390 (95% CI: 1.188–1.592) with a *p*-value of 0.001 than those with lower grades (WBC < 10 cells/HPF), which had a median score of 1.210 (95% CI: 0.979–1.441) (Figure 1e).

### 3.3. Dysregulated Urinary Proteogenomic Profile in UCC

**Figure 2:**
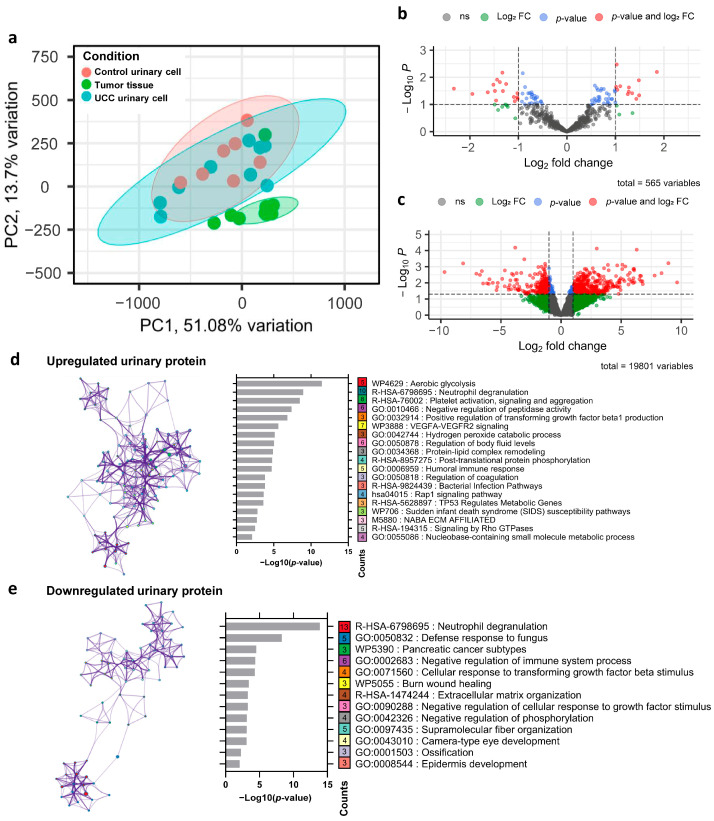
Dysregulated urinary proteogenomic profile in UCC (**a**) Principal component analysis based on transcriptomic profiles of tumor tissue, urinary cells of UCC, and urinary cells of the control group. (**b**) Volcano plot showing the protein expression differences in UCC urine compared with control urine. The dashed line represents the cut point for significance at *p*-value < 0.1 and |log2 fold change| > 1. (**c**) Volcano plot illustrating the mRNA expression differences in UCC urinary cells compared with control urinary cells. The dashed line represents the cut point for significance at *p*-value < 0.1 and |log2 fold change| > 1. (**d**) Pathways enriched in upregulated UCC urinary proteins. The left side shows the pathway network (colored nodes by pathway); the right bar graph shows the *p*-value analysis, where the number in each box indicates the gene count within that pathway. (**e**) Pathways enriched in downregulated UCC urinary proteins, displayed and explained similarly to (d).

PCA revealed a clear distinction between the transcriptomic profiles of control urine and tumor tissue. Additionally, UCC urine samples showed an overlap in transcriptomic features between control urine and UCC tumors. This aligns with the high tumor proportion observed in UCC urine cellularity, which also showed immune cell contamination (Figure 2a). To identify differentially expressed genes, we analyzed transcriptomic data from nine UCC and seven control urinary cell samples. This analysis revealed 1894 significantly dysregulated genes in the UCC urinary cells, comprising 818 upregulated and 1076 downregulated genes (Figure 2c). For protein expression analysis, proteomic data from 27 UCC urine samples and 26 control samples were initially processed for protein identification. After searching against the Swiss-Prot database, we identified 565 proteins across all samples in our cohort. Differential expression analysis revealed 85 significantly dysregulated proteins (representing 15.04% of the initially annotated proteins) in UCC patients compared to controls, shown in the volcano plot (Figure 2b). Among these, 31 proteins had |log2FC| > 1, including 13 upregulated and 18 downregulated proteins. These results suggest a distinctive proteogenomic signature characterizing UCC urine. To explore the biological significance of the protein dysregulated in UCC urine, we conducted pathway analysis on these 85 proteins. The analysis of upregulated proteins identified 19 enriched pathways (Figure 2d). Five of these pathways (26.31%) involved cell adhesion, cellular component organization, and extracellular matrix remodeling, all known to play roles in cancer invasion and metastasis. Additionally, seven pathways (15.79%) related to metabolic processes and energy production, three pathways (15.79%) to humoral immune response, and one pathway (5.26%) to angiogenesis. These pathways are well established as part of cancer hallmarks, which comprise biological functions crucial for cancer development.

In contrast, the downregulated proteins were significantly enriched in 13 pathways (Figure 2e). Among these, three pathways (23.08%) were linked to the negative regulation of various biological processes, including the immune system process, cellular response to growth factor stimuli, and phosphorylation. The decrease in proteins involved in the negative regulation of cellular responses to growth factor stimuli and phosphorylation could lead to increased activity in these pathways, which generally promotes the growth and survival of cancer cells. Interestingly, the immune-related pathway neutrophil degranulation was enriched in both the upregulated and downregulated protein sets, suggesting complex and potentially biphasic regulatory dynamics within this critical immune process.

### 3.4. Identification of Urine Biomarkers for UCC Diagnosis

**Figure 3:**
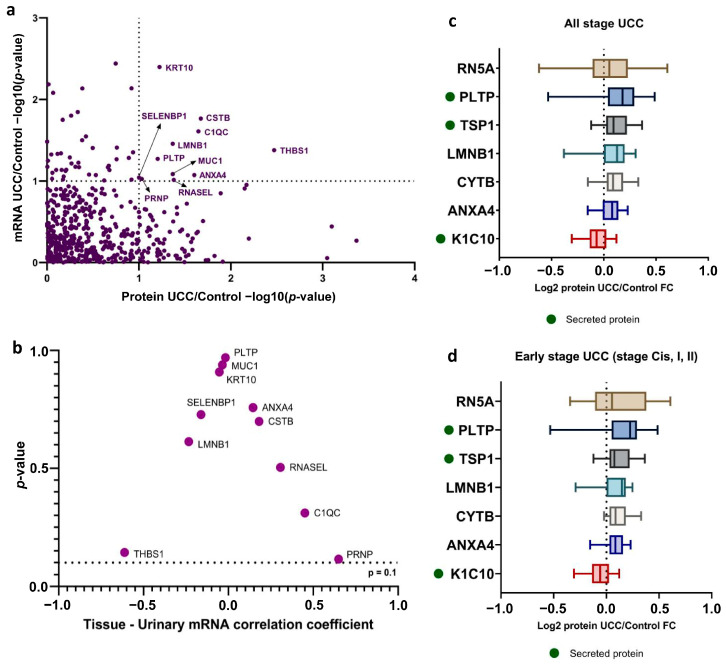
Identification of urine biomarker for UCC diagnosis (**a**) Scatterplot of the per-gene *p*-value of differential expression analysis between mRNA (*y*-axis) and protein (*x*-axis). Genes with labels are those that showed significantly dysregulated in both levels (*p*-value < 0.1). (**b**) The Spearman correlation analysis investigates the relationship between tissue-specific mRNA expression and urinary mRNA levels. (**c**) Level of significantly dysregulated protein in all UCC urine and (**d**) early-stage UCC (Cis, I, II) compared to control urine. Secreted proteins are indicated with a green dot.

To identify a urine biomarker specific to UCCs, we examined 85 proteins and 1894 mRNAs that showed significantly different expression in UCC urine. Of these, 11 proteins (12.94%) demonstrated notable dysregulation at both mRNA and protein levels, suggesting their potential as candidate biomarkers (Figure 3a, Supplementary Table S3). We then performed a correlation analysis on the genes encoding these 11 proteins. Our results showed a positive correlation between mRNA from urinary cells and tissue for five genes: *ANXA4*, *CSTB*, *RNASEL*, *C1QC*, and *PRNP*. The other six genes showed a negative correlation, though it was not statistically significant (Figure 3b). These findings support the idea that mRNA from urinary cells correlates with tumor tissue, which is a key source of mRNA and protein in urine [35]. Notably, during the early stages of UCC (stages Cis, I, and II), seven of the 11 proteins, namely RN5A, PLTP, TSP1, LMNB1, CYTB, K1C10, and ANXA4, showed similar dysregulation patterns (Supplementary Tables S3 and S4). This dysregulation was statistically significant compared to control urine samples. Among these proteins, KRT10 was downregulated, while the other six were upregulated. Additionally, PLTP and THBS1, both upregulated proteins, were identified as secreted proteins, which makes them easier to detect in urine (Figure 3c,d).

**Figure 4:**
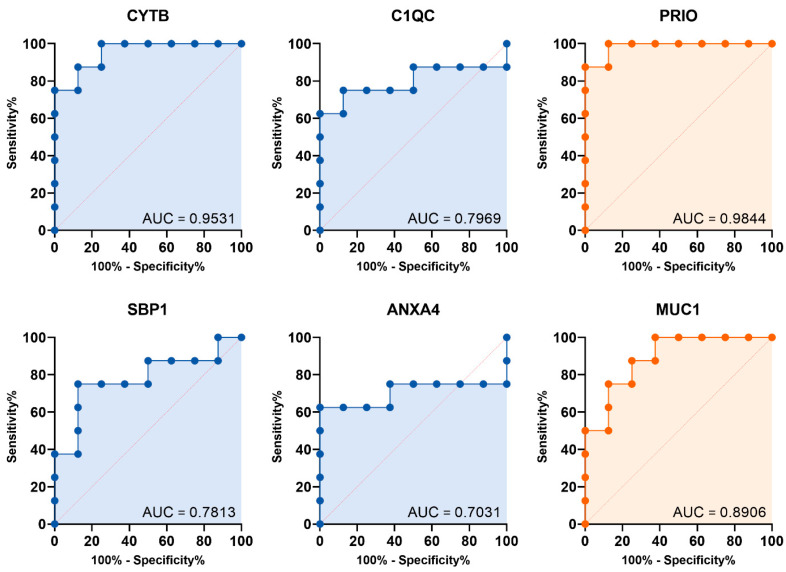
Diagnostic performance of the candidate marker: Logistic ROC curves for the identified urine protein marker, with significantly upregulated (blue) and downregulated (orange) levels in UCC.

To assess their diagnostic ability, we built a prediction model for all identified protein markers using logistic regression analysis. We then evaluated this model’s diagnostic performance with a validation dataset from the ProteomeXchange consortium, showing the ROC curve results. The area under the curve (AUC) was calculated (Figure 4). Of the 11 markers tested, six had AUC values above 0.700, which is considered acceptable. Notably, four upregulated protein markers in UCC (CYTB, C1QC, SBP1, and ANXA4) showed significant diagnostic accuracy, with AUCs of 0.9531 (95% CI: 0.8580–1.000), 0.7969 (95% CI: 0.5478–1.000), 0.7813 (95% CI: 0.5390–1.000), and 0.7031 (95% CI: 0.4040–1.000), respectively. Additionally, two downregulated proteins, PRIO and MUC1, also had strong diagnostic power, with AUCs of 0.9844 (95% CI: 0.9358–1.000) and 0.8906 (95% CI: 0.7314–1.000), respectively. For the upregulated markers, we used a Youden index-optimized cut-off: CYTB (>0.4835), C1QC (>0.5427), SBP1 (>0.5668), and ANXA4 (>0.5768). These cut-offs provided high specificity rates of 100.0%, 75.0%, 75.0%, and 62.5% for CYTB, C1QC, SBP1, and ANXA4, respectively, while the corresponding sensitivity rates were 75.0%, 87.5%, 87.5%, and 100%.

### 3.5. Urine Protein Biomarkers Identify Poor Prognosis in UCC

**Figure 5:**
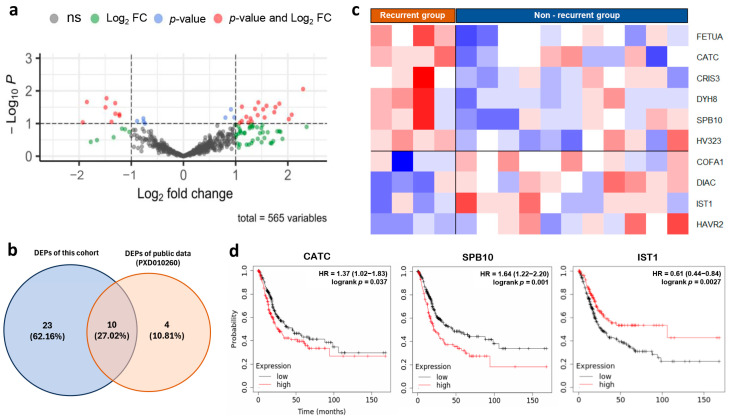
Identifying prognostic urine markers for UCC (**a**) Volcano plot illustrating the proteins significantly dysregulated in NMIBC with recurrent compared with the non-recurrent group. (**b**) Venn diagram showing the overlap of significantly dysregulated proteins identified in our discovery cohort and a validation dataset. (**c**) Heatmap representing protein expression level of 10 dysregulated proteins in NMIBC, with the recurrent compared with the non-recurrent group in both our cohort and validation dataset. (**d**) Comparative survival analysis between UCC patients with high and low expression levels of the gene marker for recurrence.

We performed differential expression analyses of the urine proteome from 4 NMIBC patients with recurrence and 11 NMIBC patients without recurrence. We successfully identified 33 dysregulated proteins in the recurrent group compared to the non-recurrent group, with |log2FC| > 1 (Figure 5a, Supplementary Table S5). To validate our findings, we analyzed differential protein expression in proteomic data from the same validation dataset, which included eight NMIBC samples. Specifically, we compared tissue protein expression in 3 NMIBC cases with recurrent disease and 5 cases without recurrence. The results showed that 14 proteins were significantly dysregulated in the recurrence group (Supplementary Table S6). Interestingly, 10 of these 14 proteins were also dysregulated in our cohort (Figure 5b,c). To explore the relationship between these candidate markers and clinical outcomes, particularly survival rates, we conducted a survival analysis using a large gene expression database [33]. This analysis showed that high expression of genes encoding two proteins, CATC and SPB10, was significantly associated with poorer survival in bladder cancer, while IST1 expression was linked to better survival (Figure 5d). This aligned with our internal cohort analysis, where patients were separated by the median expression of each protein; the high expression groups for CATC and SPB10 demonstrated significantly poorer disease-free survival, yielding a Hazard Ratio (HR) of 9.242 (95% CI: 1.116–76.550, *p*-value 0.013) and 4.997 (95% CI: 1.164–21.460, *p*-value 0.017), respectively.

## 4. Discussion

Approximately 20% of patients with UCC are diagnosed at a higher T-stage (T-stage ≥ 2), which is linked to a lower 5-year overall survival rate of about 28%, compared to 69% in those with lower T-stage disease (T < 2). Early detection of bladder cancer is crucial because it can improve patient survival and treatment outcomes [4]. A high-performance biomarker is essential for effectively detecting cancer in its early stages. Our proteogenomic approach to UCC urine samples successfully identified a group of potential biomarkers. First, we used an RNA-based cellularity inference tool to assess the cellularity and purity of the samples. The results showed that urine samples from UCC patients are a promising source for detecting tumor cells, with cellularity reaching up to 0.605. However, the urine cellularity profile exhibited significant immune cell contamination, especially in patients with higher hematuria grades, who showed significantly higher ImmuneScores, suggesting a correlation between immune cell presence and hematuria severity, likely indicating these immune cells originate from the bloodstream.

It is important to address the reliability of using urine samples for biomarker detection under conditions of hematuria, which is a common presentation in UCC. While our analysis showed that all urine samples exhibited an elevated immune cell component, suggesting cellular dilution, subsequent findings confirm that tumor-specific material is still captured effectively. Crucially, the subsequent data in our study demonstrate that our panel of protein markers remains significantly dysregulated in the UCC urine samples and performs remarkably well, confirming that sufficient tumor-derived material is successfully captured and concentrated, even in the presence of immune and blood cells. This robustness is aligned with meta-analyses that previously established the detectability of tumor-specific protein markers in bladder cancer patients with hematuria, regardless of the degree of hematuria (microscopic versus macroscopic) [36,37].

Our pathway analysis also revealed that pathways involved in cancer cell invasiveness and metastasis were upregulated in UCC urine. Conversely, most downregulated proteins were enriched in pathways related to the negative regulation of biological processes that usually support cancer growth and survival. These findings confirm the presence of cancer-related molecules in patients’ urine. Furthermore, we identified four potential protein markers: CYTB, C1QC, SBP1, and ANXA4. Notably, CYTB and ANXA4 are upregulated even in early disease stages, making them promising candidates for point-of-care (POC) testing. CYTB, encoded by the CSTB gene, inhibits cysteine proteases like cathepsins, which is vital for regulating proteolysis involved in cell proliferation, differentiation, apoptosis, and immune regulation [38]. Elevated CYTB levels in tissues and urine of bladder cancer patients are associated with tumor grade, stage, and recurrence [39,40], suggesting CSTB’s role in influencing malignant behavior through regulation of proteolysis and cellular pathways.

ANXA4, part of the annexin family that binds phospholipids in a calcium-dependent manner, plays crucial roles in cellular processes such as division and apoptosis [41]. Studies show that ANXA4 is overexpressed in bladder cancer tissues, especially in the luminal subtype [42,43], and its increased expression promotes cell cycle progression and inhibits apoptosis, correlating with poorer prognosis [44]. It likely contributes to malignancy by enhancing proliferation and resistance to cell death. SBP1, a selenium-binding protein, may act as a tumor suppressor in multiple cancers [45], with dysregulation linked to poor prognosis in bladder cancer [46]. C1QC, part of the complement system, is connected to immune responses and macrophages in the tumor microenvironment [47,48]. Despite extensive research on CYTB, ANXA4, and SBP1 in bladder cancer tissue, direct studies on C1QC’s role in bladder cancer are limited, though its immune regulatory functions suggest it could influence tumor progression.

Only CYTB has been confirmed as detectable in urine, indicating its potential as a liquid biomarker. Limited data exist on SBP1 and ANXA4 in urine samples. Our study shows these markers perform remarkably, with AUCs of 0.9531 for CYTB, 0.7969 for C1QC, 0.7813 for SBP1, and 0.7031 for ANXA4. These results are comparable to FDA-approved urine tests like NMP22 and hCFHrp, which show sensitivities of 56% and 67%, and specificities of 88% and 75%, respectively, with AUCs around 0.83 [49] and 0.75 [50]. This highlights their potential as non-invasive biomarkers, especially CYTB and ANXA4, which are detectable in the early stage. While these results highlight their potential as non-invasive biomarkers, validation with larger clinical cohorts is essential. We acknowledge that our initial discovery analysis compared UCC patients with non-cancerous hematuria patients. We recognize that comparing against a healthy urine proteome reference is necessary to more precisely delineate tumor-specific changes from general contamination. Therefore, future validation studies will incorporate samples from healthy controls to confirm the tumor-specificity of our proposed diagnostic markers and to establish appropriate clinical cutoff values.

Focusing on the challenge of high recurrence (50–70%) and progression (10–30%) rates in NMIBC [51]. We identified 14 protein markers associated with NMIBC recurrence, ten of which showed significant dysregulation in both our urine samples and tissue samples from the ProteomeXchange consortium. Validation with gene expression data demonstrated that high expression of CATC and SPB10 correlates with poorer survival, while IST1 expression is linked to better outcomes. These proteins may serve as novel prognostic markers, representing new links to UCC.

In this study, we conducted urinary transcriptomic and proteomic profiling to find urine-based biomarkers for UCC. While proteomics data are more limited than transcriptomics, proteins are functional gene products essential for cellular activity and are more practical for point-of-care tests [52,53]. To improve discovery reliability, we used integrated transcriptomic and proteomic analysis. This approach successfully identified urine-based diagnostic and prognostic markers demonstrating high diagnostic accuracy and potential clinical utility.

## 5. Conclusions

This study provides strong evidence supporting the use of urine as a reliable source for detecting UCC tumor cells. Additionally, the analysis of the urine proteomic profile offers significant insights into pathways involved in cancer. We specifically identified two potential diagnostic markers for urine-based testing, CYTB and ANXA4, which showed high diagnostic accuracy and can be detected in early-stage disease. Our investigation also revealed three urine markers (CATC, SPB10, and IST1) that may indicate the recurrence of NMIBC, although further validation is needed to confirm their utility.

## 6. Limitations of the Study

This study had several limitations. First, the use of unmatched patient samples as controls could introduce potential confounding factors. To address this, we conducted statistical comparisons of key characteristics, such as sex, age, and comorbidities, between the cancer and control groups to ensure comparable traits (Table 1). Second, our validation was limited to the protein expression dataset of UCC tissues, without including urine proteomic profiles. To enhance the relevance of the identified urine markers, further validation should involve urine samples collected from patients in a clinical setting. We also recommend that the validation employ a simple protein detection method, like enzyme-linked immunosorbent assay (ELISA), which is more feasible for development into a point-of-care test. Third, our study did not include the proteomic profile of healthy, non-hematuria urine samples in validation, which would confirm the markers’ ability to distinguish diseased individuals from healthy ones. Additionally, to improve the clinical utility of urine markers, it would be beneficial to incorporate urine proteomic profiles from other urological diseases into the validation dataset. This would allow us to evaluate their capacity to differentiate UCC from other diseases within the same organ system, which could influence the urine’s proteomic profile.

## Figures and Tables

**Table 1:** Comparison of characteristics between cohort with urinary transcriptomic and proteomic data.

Characteristics		Cohort with Urinary Cell Transcriptomic Data					Cohort with Urine Proteomic Data				
		Patients with Bladder Cancer (Cancer, *n* = 9)		Hematuria Patients with Non-Cancerous Cause (Control, *n* = 7)		*p*-Value	Patients with Bladder Cancer (Cancer, *n* = 27)		Hematuria Patients with Non-Cancerous Cause (Control, *n* = 26)		*p*-Value
		*n*	(%)	*n*	(%)		*n*	(%)	*n*	(%)	
Sex	Male	8	88.9	5	42.9	0.5500	19	70.4	21	80.8	0.7681
	Female	1	11.1	2	14.3		8	29.6	7	26.9	
Age at diagnosis	(Median, range)	74 (58–94)		66 (50–89)		0.5863	74 (50–94)		64.5 (50–89)		0.0642
Comorbidities	HT	6	66.7	3	42.9	0.6145	12	44.4	12	46.2	1.0000
	DM	3	33.3	1	14.3	0.5846	7	25.9	7	26.9	1.0000
	DLP	3	33.3	3	0.0	1.0000	12	44.4	11	42.3	1.0000
	BPH	2	22.2	1	14.3	1.0000	3	11.1	4	15.4	0.7040
	CVD	2	22.2	0	14.3	0.4750	4	14.8	2	7.7	0.6687
	CVA	0	0.0	1	42.9	0.4375	1	3.7	3	11.5	0.3507
	CKD	2	22.2	1	42.9	1.0000	3	11.1	4	15.4	0.7040
	Other	4	44.4	3	14.3	1.0000	9	33.3	7	26.9	0.7664
Histology grade	High grade	8	88.9				20	74.1			
	Low grade	1	11.1				6	22.2			
	N/A	0	0.0				1	3.7			
Type of disease	NMIBC	4	44.4				15	55.6			
	MIBC	5	55.6				11	40.7			
	N/A	0	0.0				1	3.7			

HT: hypertension; DM: diabetes; DLP: Dyslipidemia; BPH: benign prostatic hyperplasia; CVD: cardiovascular disease; CVA: cerebrovascular accident; CKD: chronic kidney disease; NMIBC: non-muscle invasive bladder cancer; MIBC: invasive bladder cancer.

## Data Availability

The RNA sequencing data generated and analyzed in the current study are available at the SRA database [PRJNA110768; https://www.ncbi.nlm.nih.gov/bioproject/PRJNA1107683 (accessed on 2 December 2025); Release on 4 December 2025]. For proteomic data, the raw quantity of relative protein abundance is available in the Supplementary Material.

## Supplementary Materials

The following supporting information can be downloaded at: https://www.mdpi.com/article/10.3390/biomedicines13123020/s1, Supplementary Figure S1: Study workflow; Supplementary Tables S1: Summary of clinicopathological information of the cohort; Supplementary Table S2: Urine and tumor cellularity; Supplementary Table S3: The p-values and log2 fold changes for genes significantly dysregulated at both the mRNA and protein levels; Supplementary Table S4: Statistic value of dysregulated proteins in early-stage UCC comparing to control group; Supplementary Table S5: Statistic value of dysregulated proteins in the recurrent group of NMIBC compared to the non-recurrent group of our cohort; Supplementary Table S6: Statistic value of dysregulated proteins in the recurrent group of NMIBC compared to the non-recurrent group of validation dataset (PXD010260); Supplementary Material: relative protein abundance.

## References

[1] Sung H., Ferlay J., Siegel R.L., Laversanne M., Soerjomataram I., Jemal A., Bray F. (2021). Global Cancer Statistics 2020: GLOBOCAN Estimates of Incidence and Mortality Worldwide for 36 Cancers in 185 Countries. CA Cancer J. Clin..

[2] Antoni S., Ferlay J., Soerjomataram I., Znaor A., Jemal A., Bray F. (2017). Bladder Cancer Incidence and Mortality: A Global Overview and Recent Trends. Eur. Urol..

[3] Santoni G., Morelli M.B., Amantini C., Battelli N. (2018). Urinary Markers in Bladder Cancer: An Update. Front. Oncol..

[4] Zang Y., Li X., Cheng Y., Qi F., Yang N. (2020). An overview of patients with urothelial bladder cancer over the past two decades: A Surveillance, Epidemiology, and End Results (SEER) study. Ann. Transl. Med..

[5] International Collaboration of Trialists on behalf of the Medical Research Council Advanced Bladder Cancer Working Party (now the National Cancer Research Institute Bladder Cancer Clinical Studies Group), The European Organisation for Research and Treatment of Cancer Genito-Urinary Tract Cancer Group, The Australian Bladder Cancer Study Group, The National Cancer Institute of Canada Clinical Trials Group, Finnbladder, Norwegian Bladder Cancer Study Group, Club Urologico Espanol de Tratamiento Oncologico Group (2011). International phase III trial assessing neoadjuvant cisplatin, methotrexate, and vinblastine chemotherapy for muscle-invasive bladder cancer: Long-term results of the BA06 30894 trial. J. Clin. Oncol..

[6] Matuszczak M., Kiljanczyk A., Salagierski M. (2022). A Liquid Biopsy in Bladder Cancer-The Current Landscape in Urinary Biomarkers. Int. J. Mol. Sci..

[7] Crocetto F., Amicuzi U., Musone M., Magliocchetti M., Di Lieto D., Tammaro S., Pastore A.L., Fuschi A., Falabella R., Ferro M. (2025). Liquid Biopsy: Current advancements in clinical practice for bladder cancer. J. Liq. Biopsy.

[8] Chen C.K., Liao J., Li M.S., Khoo B.L. (2020). Urine biopsy technologies: Cancer and beyond. Theranostics.

[9] Maas M., Todenhofer T., Black P.C. (2023). Urine biomarkers in bladder cancer—Current status and future perspectives. Nat. Rev. Urol..

[10] Batista R., Vinagre N., Meireles S., Vinagre J., Prazeres H., Leao R., Maximo V., Soares P. (2020). Biomarkers for Bladder Cancer Diagnosis and Surveillance: A Comprehensive Review. Diagnostics.

[11] Holzbeierlein J.M., Bixler B.R., Buckley D.I., Chang S.S., Holmes R., James A.C., Kirkby E., McKiernan J.M., Schuckman A.K. (2024). Diagnosis and Treatment of Non-Muscle Invasive Bladder Cancer: AUA/SUO Guideline: 2024 Amendment. J. Urol..

[12] Babjuk M., Burger M., Capoun O., Cohen D., Comperat E.M., Dominguez Escrig J.L., Gontero P., Liedberg F., Masson-Lecomte A., Mostafid A.H. (2022). European Association of Urology Guidelines on Non-muscle-invasive Bladder Cancer (Ta, T1, and Carcinoma in Situ). Eur. Urol..

[13] Livshits M.A., Khomyakova E., Evtushenko E.G., Lazarev V.N., Kulemin N.A., Semina S.E., Generozov E.V., Govorun V.M. (2015). Isolation of exosomes by differential centrifugation: Theoretical analysis of a commonly used protocol. Sci. Rep..

[14] Afkarian M., Bhasin M., Dillon S.T., Guerrero M.C., Nelson R.G., Knowler W.C., Thadhani R., Libermann T.A. (2010). Optimizing a proteomics platform for urine biomarker discovery. Mol. Cell. Proteom..

[15] Pertea M., Kim D., Pertea G.M., Leek J.T., Salzberg S.L. (2016). Transcript-level expression analysis of RNA-seq experiments with HISAT, StringTie and Ballgown. Nat. Protoc..

[16] Andrews S. FastQC: A Quality Control Tool for High Throughput Sequence Data. http://www.bioinformatics.babraham.ac.uk/projects/fastqc.

[17] Bolger A.M., Lohse M., Usadel B. (2014). Trimmomatic: A flexible trimmer for Illumina sequence data. Bioinformatics.

[18] Kim D., Paggi J.M., Park C., Bennett C., Salzberg S.L. (2019). Graph-based genome alignment and genotyping with HISAT2 and HISAT-genotype. Nat. Biotechnol..

[19] Li H., Handsaker B., Wysoker A., Fennell T., Ruan J., Homer N., Marth G., Abecasis G., Durbin R., 1000 Genome Project Data Processing Subgroup (2009). The Sequence Alignment/Map format and SAMtools. Bioinformatics.

[20] Pertea M., Pertea G.M., Antonescu C.M., Chang T.C., Mendell J.T., Salzberg S.L. (2015). StringTie enables improved reconstruction of a transcriptome from RNA-seq reads. Nat. Biotechnol..

[21] Yoshihara K., Shahmoradgoli M., Martinez E., Vegesna R., Kim H., Torres-Garcia W., Trevino V., Shen H., Laird P.W., Levine D.A. (2013). Inferring tumour purity and stromal and immune cell admixture from expression data. Nat. Commun..

[22] Aran D., Hu Z., Butte A.J. (2017). xCell: Digitally portraying the tissue cellular heterogeneity landscape. Genome Biol..

[23] Rappsilber J., Mann M., Ishihama Y. (2007). Protocol for micro-purification, enrichment, pre-fractionation and storage of peptides for proteomics using StageTips. Nat. Protoc..

[24] Gillet L.C., Navarro P., Tate S., Rost H., Selevsek N., Reiter L., Bonner R., Aebersold R. (2012). Targeted data extraction of the MS/MS spectra generated by data-independent acquisition: A new concept for consistent and accurate proteome analysis. Mol. Cell. Proteom..

[25] Vitting-Seerup K., Sandelin A. (2019). IsoformSwitchAnalyzeR: Analysis of changes in genome-wide patterns of alternative splicing and its functional consequences. Bioinformatics.

[26] Robinson M.D., McCarthy D.J., Smyth G.K. (2010). edgeR: A Bioconductor package for differential expression analysis of digital gene expression data. Bioinformatics.

[27] Ritchie M.E., Phipson B., Wu D., Hu Y., Law C.W., Shi W., Smyth G.K. (2015). limma powers differential expression analyses for RNA-sequencing and microarray studies. Nucleic Acids Res..

[28] Zhou Y., Zhou B., Pache L., Chang M., Khodabakhshi A.H., Tanaseichuk O., Benner C., Chanda S.K. (2019). Metascape provides a biologist-oriented resource for the analysis of systems-level datasets. Nat. Commun..

[29] Shannon P., Markiel A., Ozier O., Baliga N.S., Wang J.T., Ramage D., Amin N., Schwikowski B., Ideker T. (2003). Cytoscape: A software environment for integrated models of biomolecular interaction networks. Genome Res..

[30] Aldousari S., Kassouf W. (2010). Update on the management of non-muscle invasive bladder cancer. Can. Urol. Assoc. J..

[31] Deutsch E.W., Bandeira N., Perez-Riverol Y., Sharma V., Carver J.J., Mendoza L., Kundu D.J., Wang S., Bandla C., Kamatchinathan S. (2023). The ProteomeXchange consortium at 10 years: 2023 update. Nucleic Acids Res..

[32] Berle M., Ghila L., Vethe H., Chaudhry A., Garberg H., Beisland C., Haaland O.A., Oveland E., Halvorsen O.J., Davidsson T. (2018). Novel protein signatures suggest progression to muscular invasiveness in bladder cancer. PLoS ONE.

[33] Nagy A., Munkacsy G., Gyorffy B. (2021). Pancancer survival analysis of cancer hallmark genes. Sci. Rep..

[34] van Hoogstraten L.M.C., Vrieling A., van der Heijden A.G., Kogevinas M., Richters A., Kiemeney L.A. (2023). Global trends in the epidemiology of bladder cancer: Challenges for public health and clinical practice. Nat. Rev. Clin. Oncol..

[35] Bryan R.T., Regan H.L., Pirrie S.J., Devall A.J., Cheng K.K., Zeegers M.P., James N.D., Knowles M.A., Ward D.G. (2015). Protein shedding in urothelial bladder cancer: Prognostic implications of soluble urinary EGFR and EpCAM. Br. J. Cancer.

[36] Sathianathen N.J., Butaney M., Weight C.J., Kumar R., Konety B.R. (2018). Urinary Biomarkers in the Evaluation of Primary Hematuria: A Systematic Review and Meta-Analysis. Bladder Cancer.

[37] Soputro N.A., Gracias D.N., Dias B.H., Nzenza T., O’Connell H., Sethi K. (2022). Utility of urinary biomarkers in primary haematuria: Systematic review and meta-analysis. BJUI Compass.

[38] Ye D., Duan X., Guan B., Yuan J., Zhu Y., Shi J., Lu Q., Xu G. (2024). Biomarker cystatin B expression correlates with pathogenesis in cervical cancer. J. Int. Med. Res..

[39] Huang C., Ai X., Hu L., Ren D. (2022). The Role of NMP22 and CSTB Levels in Predicting Postoperative Recurrence of Bladder Cancer. J. Immunol. Res..

[40] Feldman A.S., Banyard J., Wu C.L., McDougal W.S., Zetter B.R. (2009). Cystatin B as a tissue and urinary biomarker of bladder cancer recurrence and disease progression. Clin. Cancer Res..

[41] Yao H., Sun C., Hu Z., Wang W. (2016). The role of annexin A4 in cancer. Front. Biosci. (Landmark Ed.).

[42] Shen C., Zhang S., Zhang Z., Yang S., Zhang Y., Lin Y., Fu C., Li Z., Wu Z., Wang Z. (2023). Pan-cancer evidence of prognosis, immune infiltration, and immunotherapy efficacy for annexin family using multi-omics data. Funct. Integr. Genom..

[43] Wu W., Jia G., Chen L., Liu H., Xia S. (2021). Analysis of the Expression and Prognostic Value of Annexin Family Proteins in Bladder Cancer. Front. Genet..

[44] Liu J., Wang H., Zheng M., Deng L., Zhang X., Lin B. (2020). p53 and ANXA4/NF-kappaB p50 complexes regulate cell proliferation, apoptosis and tumor progression in ovarian clear cell carcinoma. Int. J. Mol. Med..

[45] Yang W., Diamond A.M. (2013). Selenium-binding protein 1 as a tumor suppressor and a prognostic indicator of clinical outcome. Biomark. Res..

[46] Wang Y., Zhu W., Chen X., Wei G., Jiang G., Zhang G. (2020). Selenium-binding protein 1 transcriptionally activates p21 expression via p53-independent mechanism and its frequent reduction associates with poor prognosis in bladder cancer. J. Transl. Med..

[47] Yao W., Liu H., Xu F., Cai Z., Hang L., Lu M., Zhao Y., Yang C., Zong Y. (2023). C1QC is a prognostic biomarker with immune-related value in kidney renal clear cell carcinoma. Front. Genet..

[48] Chen L.H., Liu J.F., Lu Y., He X.Y., Zhang C., Zhou H.H. (2021). Complement C1q (C1qA, C1qB, and C1qC) May Be a Potential Prognostic Factor and an Index of Tumor Microenvironment Remodeling in Osteosarcoma. Front. Oncol..

[49] Wang Z., Que H., Suo C., Han Z., Tao J., Huang Z., Ju X., Tan R., Gu M. (2017). Evaluation of the NMP22 BladderChek test for detecting bladder cancer: A systematic review and meta-analysis. Oncotarget.

[50] Guo A., Wang X., Gao L., Shi J., Sun C., Wan Z. (2014). Bladder tumour antigen (BTA stat) test compared to the urine cytology in the diagnosis of bladder cancer: A meta-analysis. Can. Urol. Assoc. J..

[51] Bree K.K., Shan Y., Hensley P.J., Lobo N., Hu C., Tyler D.S., Chamie K., Kamat A.M., Williams S.B. (2022). Management, Surveillance Patterns, and Costs Associated with Low-Grade Papillary Stage Ta Non-Muscle-Invasive Bladder Cancer Among Older Adults, 2004–2013. JAMA Netw. Open.

[52] Loktionov A. (2020). Biomarkers for detecting colorectal cancer non-invasively: DNA, RNA or proteins?. World J. Gastrointest. Oncol..

[53] Ng C.K.Y., Dazert E., Boldanova T., Coto-Llerena M., Nuciforo S., Ercan C., Suslov A., Meier M.A., Bock T., Schmidt A. (2022). Integrative proteogenomic characterization of hepatocellular carcinoma across etiologies and stages. Nat. Commun..

