# Supplementary material for: Integrated Proteogenomic Approach for Discovering Potential Biomarkers in Urothelial Carcinoma of the Bladder
Source: Biomedicines. 2025 Dec 10;13(12):3020. doi: 10.3390/biomedicines13123020 (PMC12730745; doi:10.3390/biomedicines13123020)
Supplement: Supplementary file 1 [file biomedicines-13-03020-s001.zip › Supplementary table S1-S6_ed1.pdf]

**Supplementary Table S1** Summary of clinicopathological information of the cohort					
Characteristics		Patients with UCC (Cancer, n=41)		Hematuria patients with non-cancerous cause (Control, n=27)	
		n	(%)	n	(%)
Sex	Male	30	73.2	22	81.5
	Female	11	26.8	5	18.5
Age at diagnosis	(Median, range)	74 (42-94)		64 (50-89)	
Comorbidities	HT	17	41.5	12	44.4
	DM	10	24.4	7	25.9
	DLP	16	39.0	11	40.7
	BPH	6	14.6	4	14.8
	CVD	5	12.2	2	7.4
	CVA	2	4.9	3	11.1
	CKD	4	9.8	4	14.8
	Other	12	29.3	8	29.6
Tumor size	< 3 cm	25	61.0		
	≥ 3 cm	16	39.0		
Histology grade	High grade	28	68.3		
	Low grade	12	29.3		
	N/A	1	2.4		
Type of disease	NMIBC	25	61.0		
	MIBC	14	34.1		
	N/A	2	4.9		
Staging	Cis	16	39.0		
	I	8	19.5		
	II	5	12.2		
	III	6	14.6		
	IV	4	9.8		
	N/A	2	4.9		
Recurrence	Yes	9	22.0		
	No	32	78.0		
Current status	Alive	28	68.3		
	Dead	13	31.7		

Statistical comparisons were performed using Fisher's exact tests (for categorical data), and two-sided Wilcoxon Rank Sum Test (for numerical and ordinal data). HT = Hypertension, DM = Diabetes mellitus, DLP = Dyslipidemia, BPH = Benign prostatic hyperplasia, CVD = Cardiovascular disease, CVA = Cerebrovascular accident, and CKD = Chronic kidney disease.

Supplementary table S2 Urine and tumor cellularity

Sample type	Sample ID	Estimated proportion of tumor cell (ESTIMATE)	ImmuneScore (xCell)	StromaScore (xCell)	Microenvironment Score (xCell)
Cancer urinary cell (UCC)	BC31u	0.5942	1.2135	0.1442	1.3577
	BC33u	0.4349	1.9309	0	1.9309
	BC38u	0.5321	1.3237	0.0264	1.3501
	BC42u	0.5965	1.3979	0	1.3979
	BC43u	0.6443	1.2473	0	1.2473
	BC44u	0.8597	0.6838	0	0.6838
	BC46u	0.5088	1.2852	0.077	1.3622
	BC51u	0.7496	1.1615	0	1.1615
	BC54u	0.5312	1.6737	0	1.6737
Tumor tissue	BC01t	0.6304	0.9927	0.1621	1.1548
	BC03t	0.9848	0.4580	0.1106	0.5686
	BC04t	0.9973	0.3412	0.0316	0.3728
	BC05t	0.9709	0.3311	0.0758	0.407
	BC07t	0.9936	0.3513	0.0172	0.3685
	BC08t	0.9866	0.2782	0.0722	0.3504
	BC09t	0.9726	0.3947	0.0772	0.4719
	BC10t	0.9949	0.3464	0.0757	0.4221
	BC11t	0.9888	0.2223	0.0902	0.3126
	BC17t	0.9926	0.3873	0.0786	0.4659
	BC19t	0.9876	0.3125	0.0816	0.3941
	BC28t	0.9893	0.3446	0.1478	0.4924
	BC29t	0.9702	0.4689	0.0492	0.5181
	BC31t	0.9860	0.2165	0.0694	0.2859
	BC40t	0.9999	0.3718	0.0534	0.4252
Normal urinary cell	NC02	-	0.8079	0.0822	0.8901
	NC03	-	1.9024	0	1.9024
	NC11	-	1.9937	0	1.9937
	NC22	-	1.7423	0	1.7423
	NC24	-	1.3502	0	1.3502
	NC34	-	1.2368	0	1.2368
	NC36	-	1.3945	0.0653	1.4598
	NC38	-	1.0496	0	1.0496

Supplementary table S3 The p-values and log2 fold changes for genes significantly dysregulated at both the mRNA and protein levels

Uniprot_ID	Gene_name	log2FC_Protein	log10(Pval_Protein)	log2FC_RNA	log10(Pval_RNA)
P07996	THBS1	1.0263	-2.4711	-1.7408	-1.3786
P04080	CSTB	1.0996	-1.6725	2.9420	-1.7656
P02747	C1QC	1.0136	-1.6464	1.7574	-1.6098
P09525	ANXA4	0.6179	-1.6023	0.8078	-1.0729
Q05823	RNASEL	1.2550	-1.3791	-0.8544	-1.0150
P20700	LMNB1	0.8672	-1.3675	-1.5827	-1.4568
P15941	MUC1	-0.7250	-1.3670	-1.8498	-1.0850
P13645	KRT10	-0.6870	-1.2228	2.0693	-2.3983
P55058	PLTP	0.9318	-1.2036	1.5292	-1.2716
P04156	PRNP	-0.5127	-1.0305	1.2389	-1.0266
Q13228	SELENBP1	0.5065	-1.0000	1.1967	-1.0425

Supplementary table S4 Statistic value of dysregulated proteins in early-stage UCC comparing to control group

Prot_name	Uniprot_ID	logFC	AveExpr	P.Value
TSP1	P07996	1.0645	13.5927	0.0059
ANXA4	P09525	0.7607	14.1090	0.0123
RN5A	Q05823	1.5175	10.4115	0.0258
CYTB	P04080	1.1006	10.4128	0.0368
LMNB1	P20700	0.9442	11.4712	0.0463
PLTP	P55058	0.9764	9.2047	0.0778
K1C10	P13645	-0.6703	15.7855	0.0965

Supplementary table S5 Statistic value of dysregulated proteins in the recurrent group of NMIBC compared to the non-recurrent group of our cohort

Prot_name	Uniprot_ID	logFC	AveExpr	P.Value
DYH8	Q96JB1	2.7133	13.1376	0.0068
FETUA	P02765	1.4130	16.2893	0.0264
CRIS3	P54108	1.6432	11.4918	0.0550
A1BG	P04217	1.4404	15.8800	0.0730
SPB10	P48595	1.2458	13.1738	0.0779
CATC	P53634	1.9687	14.1821	0.0812
ZA2G	P25311	1.7532	17.0941	0.0829
RHG10	A1A4S6	4.7728	7.1886	0.0849
DLL1	O00548	1.7748	12.1984	0.0911
HV323	P01764	1.8463	13.5146	0.0939
DIAC	Q01459	-2.2623	11.7144	0.0096
DNAI3	Q8IWG1	-1.8665	11.1159	0.0137
COCA1	Q99715	-2.0528	10.8219	0.0144
LYAG	P10253	-1.4422	13.8955	0.0270
LBP	P18428	-2.1763	11.9639	0.0285
COFA1	P39059	-1.8363	10.6034	0.0356
ITIH3	Q06033	-1.6846	13.6189	0.0365
KIF28	B7ZC32	-2.0849	10.8148	0.0367
HAVR2	Q8TDQ0	-2.0722	11.5349	0.0458
IGKC	P01834	-1.0841	12.9987	0.0506
HS71B	P0DMV9	-1.1056	12.0884	0.0530
POSTN	Q15063	-1.1764	10.9159	0.0566
ANXA3	P12429	-1.4335	11.3888	0.0714
MUCL1	Q96DR8	-1.3611	13.0355	0.0729
HV307	P01780	-1.4915	11.6068	0.0732
LAIR1	Q6GTX8	-1.6043	11.5384	0.0762
FABP4	P15090	-1.3057	12.0649	0.0801
IST1	P53990	-1.2706	12.2274	0.0806
APOC1	P02654	-1.2063	13.0379	0.0846
CLUS	P10909	-1.0005	14.7350	0.0852
PLSI	Q14651	-1.5739	11.5950	0.0853
UGPA	Q16851	-1.5135	10.9041	0.0861
SEPP1	P49908	-1.2947	9.4504	0.0935

Supplementary table S6 Statistic value of dysregulated proteins in the recurrent group of NMIBC compared to the non-recurrent group of validation dataset (PXD010260)

Prot_name	Uniprot_ID	logFC	AveExpr	P.Value
DYH8*	Q96JB1	2.2925	13.3094	0.0088
COFA1*	P39059	-1.4740	10.7833	0.0169
HAVR2*	Q8TDQ0	-1.8504	11.4230	0.0219
BLVRB	P30043	1.4351	11.2873	0.0225
IST1*	P53990	-1.3075	12.4002	0.0237
CRIS3*	P54108	1.8381	10.9030	0.0246
S10A8	P05109	1.5913	16.4942	0.0288
FETUA*	P02765	1.1196	16.1095	0.0306
CATC*	P53634	1.7622	14.2132	0.0315
DIAC*	Q01459	-1.4935	11.9980	0.0320
SPB10*	P48595	1.3712	13.1322	0.0356
CO1A1	P02452	1.5270	14.4736	0.0370
ITIH2	P19823	0.9098	14.8593	0.0370
HV323*	P01764	1.7403	13.8100	0.0449

*These proteins also exhibited dysregulated expression in the recurrent group of NMIBC within our cohort
